# Education Is Positively and Causally Linked With Spatial Navigation Ability Across the Lifespan

**DOI:** 10.1162/opmi.a.13

**Published:** 2025-07-26

**Authors:** Antoine Coutrot, Rogier A. Kievit, Stuart J. Ritchie, Ed Manley, Jan M. Wiener, Christof Hölscher, Ruth C. Dalton, Michael Hornberger, Hugo J. Spiers

**Affiliations:** CNRS, INSA Lyon, Universite Claude Bernard Lyon 1, LIRIS, UMR 5205, Villeurbanne, France; Cognitive Neuroscience Department, Donders Institute for Brain, Cognition and Behavior, Radboud University Medical Center, Nijmegen, The Netherlands; MRC Cognition and Brain Sciences Unit, University of Cambridge, Cambridge, UK; Social, Genetic and Developmental Psychiatry Centre, King’s College London, London, UK; Centre for Advanced Spatial Analysis, University College London, London, UK; School of Geography, University of Leeds, Leeds, UK; Department of Psychology, Ageing and Dementia Research Centre, Bournemouth University, Poole, UK; ETH Zürich, Swiss Federal Institute of Technology, Zürich, Switzerland; School of Architecture, Lancaster University, Lancaster, UK; Norwich Medical School, University of East Anglia, Norwich, UK; Institute of Behavioural Neuroscience, Department of Experimental Psychology, Division of Psychology and Language Sciences, University College London, London, UK

**Keywords:** spatial ability, education, cross-country, natural experiment

## Abstract

There is consistent evidence for a positive association between education and a wide range of cognitive abilities. In particular, spatial abilities have been shown to be strongly related to academic achievement. However, studying this association is complex as both education and spatial abilities are modulated by multivariate sociodemographic factors, likely to vary across countries. Most previous studies relied on small sample sizes or were restricted to a limited number of countries, thus were unable to control for these covariates. To overcome these limitations, we used a spatial navigation task embedded in a mobile video game. We quantified the wayfinding ability of 397,162 people across 38 countries and showed that on average, education level was positively associated with wayfinding ability. This difference was stronger in older participants and increased with task difficulty. However, the effect of education was different across countries, from near-zero and non-significant in India (Bayes’ factor = 0.08, Hedge’s *g* = −0.03, 95% CI = [−0.15, 0.08]), to modest and significant in Romania (Bayes’ factor = 345.44, Hedge’s *g* = 0.15, 95% CI = [0.08, 0.22]). We did not find any relationship between the education effect size of countries and economic indicators such as GDP per capita. Using the 1972 reform increasing the minimum school leaving age in the UK as a natural experiment, we used a regression discontinuity design to show that education has a causal effect on wayfinding ability.

## INTRODUCTION

Spatial navigation is a multifaceted cognitive ability that has been strongly associated with various life outcomes, including academic achievement and career success (Buckley et al., 2018; Tosto et al., 2014; Xie et al., 2020). For instance, a longitudinal study by Tian et al. (2023) demonstrated that spatial skills assessed in fourth grade significantly predicted the likelihood of choosing a Science, Technology, Engineering and Mathematics (STEM) major in college, even after accounting for other cognitive and motivational factors (Tian et al., 2023). Despite accumulating evidence highlighting the importance of spatial ability, establishing a causal link between education level and spatial skills remains challenging. This complexity is partly due to the numerous factors intertwined with both spatial ability and educational attainment (Broer et al., 2019; Coutrot, Manley, et al., 2022; Guerra-Carrillo et al., 2017; Hackman et al., 2010; Leonard et al., 2015; Lövdén et al., 2020; Ritchie & Tucker-Drob, 2018). Socioeconomic status (SES), in particular, has been shown to influence cognitive development from an early age (Johnson et al., 2022). Disparities in SES have been associated with differences in spatial skills among preschool-aged children, with executive function playing a mediating role (Bachman et al., 2022). This suggests that children from lower SES backgrounds may have fewer opportunities to develop essential spatial skills, potentially hampering their future academic performance. Addressing these disparities through targeted educational interventions has shown promise. Spatial skills are malleable and respond positively to life experiences, and educational interventions (Baenninger & Newcombe, 1989; Wright et al., 2008). While this research emphasizes the influence of personal experience on spatial ability development, educational interventions improve spatial skills have been shown to be effective (Uttal et al., 2013). Dong et al. (2023) demonstrated that geography education can enhance students’ spatial abilities (Dong et al., 2023). Their study showed that after four years of geography education, students exhibited improved mental rotation, spatial visualization, and spatial relation reasoning abilities compared to their peers without such education. Despite these insights, most previous studies have relied on small sample sizes or have been limited to specific regions, often focusing on WEIRD (Western, Educated, Industrialized, Rich, and Democratic) populations, see Henrich et al. (2010). This limitation hampers the generalizability of findings to more diverse populations.

To overcome these limitations, we used the Sea Hero Quest database, which contains the spatial trajectories of 3.9 million participants recorded while performing a spatial navigation task in a mobile video game, ‘Sea Hero Quest’ (SHQ) (Coutrot et al., 2018; Spiers et al., 2023). SHQ was designed to quantify the player’s sense of direction, and gathered demographic information such as their age, gender, nationality or level of education. Performance at SHQ has been shown to be predictive of real-world navigation ability (Coutrot et al., 2019; Goodroe et al., 2025), and have been associated with many sociodemographic variables such as age, gender, nationality (Coutrot et al., 2018), reported sleep duration (Coutrot, Lazar, et al., 2022), upbringing environment (Coutrot, Manley, et al., 2022), starting driving age (Yavuz, Manley, et al., 2024), video gaming experience (Yavuz, He, et al., 2024), and country-level indicators such as the Gross Domestic Product per capita (Coutrot et al., 2018; Gilles et al., 2024).

## RESULTS AND DISCUSSION

We focus on SHQ wayfinding levels, where the player is asked to memorize a map of an aquatic environment with a set of ordered checkpoint 1. After the map disappears, the players have to navigate as quickly as possible to the checkpoints, in the correct order. The first two levels are tutorial levels as no sense of direction is required. To quantify spatial ability, we used the length of the trajectories in different levels: the shorter the trajectory, the better the spatial ability. Participant inclusion criteria were the same as in Coutrot, Manley, et al. (2022). First, to provide a reliable estimate of spatial navigation ability, we only included participants who had completed a minimum of eleven levels of the game (including 4 wayfinding levels), and who entered all their demographics. Then, we removed participants above 70 years old because we have previously shown a strong selection bias in this group causing their performance to be substantially higher than would be expected in unselected participants of the same age (Coutrot et al., 2018). Finally, we removed participants from countries with fewer than 500 players, or with the education classes more than 10-fold imbalanced. This resulted in 397,162 participants from 38 countries included in our analysis. Among them, there were 212,143 males (mean age: 37.81 ± 13.59 years old) and 185,173 females (mean age: 38.67 ± 14.92 years old). The levels of education were: university (42%, *N* = 166,714), college (28%, *N* = 111,463), high-school (27%, *N* = 107,849), and no formal (3%, *N* = 11,290). We merged ‘university’ and ‘college’ into a unique ‘tertiary education’ level (70%, *N* = 278,177). This was notably motivated by the fact that ‘university’ and ‘college’ have different meanings in different countries. For instance, the word ‘college’ can refer to a community college, technical school, or liberal arts college in some countries, or used interchangeably with ‘university’ in others. Similarly, we merged ‘high-school’ and ‘no formal’ into a unique’secondary education and lower’ level (30%, *N* = 119,139). We chose not to analyse separately the ‘no formal’ group because its relative low sample size makes it more liable to selection bias: people with no formal education playing a videogame for academic research might not be representative of their demographic group. To quantify wayfinding ability, we defined the “wayfinding performance” metric (*WF*), which assesses the optimality of participants’ trajectories in the wayfinding levels, while correcting for video-gaming skills (see Methods).

### Association of Age, Gender, and Education With Wayfinding Ability

A multivariate linear regression was calculated to predict wayfinding performance based on age, gender, and education and their interactions. We then used an ANOVA to summarize the main effects of the regression. Age ranged from 19 to 70 years, we used two categories for gender (males, females), and education (secondary and lower, tertiary). Age had the strongest association with performance (*F*_1,397154_ = 60884, *p* < 0.001, Pearson’s correlation *r* = −0.36, Hedge’s *g* between 19 y.o. and 70 y.o. *g* = 1.78), followed by gender (*F*_1,397154_ = 20257, *p* < 0.001, Hedge’s *g* = 0.44), and education (*F*_1,397154_ = 1665.30, *p* < 0.001, Hedge’s *g* = 0.14). There was a significant interaction between age and gender (*F*_1,397154_ = 2027.10, *p* < 0.001), between age and education (*F*_1,397154_ = 183.22, *p* < 0.001), between gender and education (*F*_1,397154_ = 76.64, *p* < 0.001), and between age, gender and education (*F*_1,397154_ = 9.92, *p* = 0.001). These interactions indicate that the effects of gender and education on wayfinding ability both increase with age, and that the effect of education is stronger in females. Figure 2a represents the associations of gender, age, and education with *WF*. We replicated previous studies showing that wayfinding performance decreases with age (Klencklen et al., 2012; Lester et al., 2017), and that males performed better than females (Nazareth et al., 2019). In previous analyses we have shown this gender difference is correlated across countries with the gender gap index across health, education, economy, and politics, as measured annually by the World Economic Forum (Coutrot et al., 2018). Here, we found that a higher level of education was positively associated with wayfinding performance, and that this association becomes in general stronger with increasing age, until 65 years old. Figure 2b represents the evolution across age of the effect size of the education—Wayfinding Performance association, quantified with Hedge’s *g* computed within 5-year windows. Hedge’s *g* increased from *g* = 0.06, 95% CI = [0.05, 0.08] at 20 years old to *g* = 0.14, 95% CI = [0.11, 0.17] at 65 years old. To interpret the increase of the effect of education with age, it is also important to consider that achieving the same amount of education around 1960 means something quite different, and more pronounced, than for people born later, with a broader access to education. In participants aged 70 years-old, the education effect size was drastically smaller, probably due to a higher Wayfinding Performance in participants of this age who had secondary education only. This performance difference is plausibly due to an earlier selection bias in this group, with older participants with less education being less likely to participate in this study (Wagner et al., 2010). We also ran the same analysis with four levels of education (university, college, high-school, no-formal), which lead to consistent results, see Supplementary Analysis and Figures S2 and S3.

**Figure 1.**
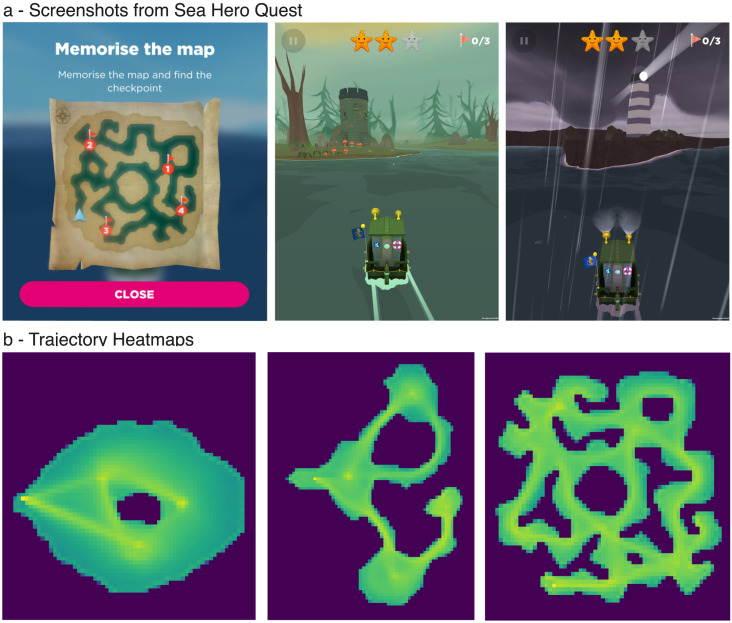
**Sea Hero Quest: a spatial navigation video game.** (a) Screenshots from example wayfinding levels. At the beginning of each wayfinding level, players must memorize the map and the location of checkpoints in a set order. The map disappears and players must steer their boat as quickly as possible to each checkpoint (see Methods). (b) Trajectory heatmaps of three wayfinding levels of different difficulty. Brighter areas correspond to the most-traveled routes.

**Figure 2.**
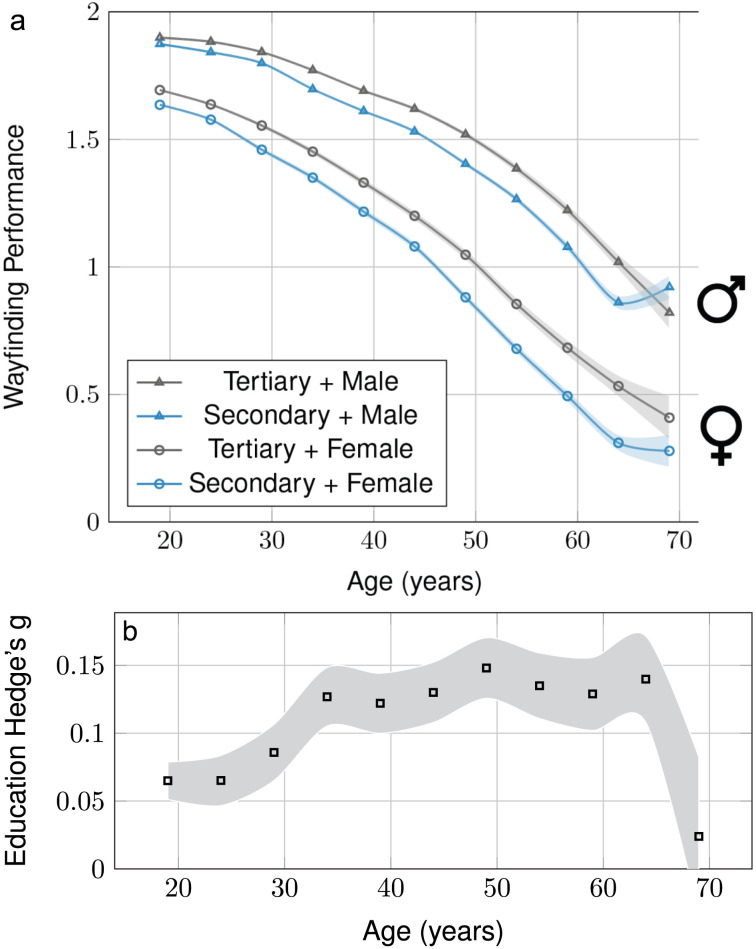
**Effect of education on Wayfinding Performance—Interaction with age and gender.** (a) Interaction with age and gender (212,143 males and 185,173 females). (b) Effect size (Hedge’s *g*) of education across age, positive values correspond to an advantage of tertiary education. Time series have been averaged within 5-year windows and error bars correspond to the 95% confidence intervals.

### Associations of Education With Wayfinding Ability Across Countries

To assess whether the associations with education are stable across countries, we fit a Linear Mixed Model (LMM) predicting wayfinding performance, with fixed effects for age and gender, and a random effect for country, with random slopes for education clustered by country. The model can be represented in the following shorthand:

$$WF∼\text{age}+\text{gender}+\left(1+\text{education}/\text{country}\right).$$

**Figure 3.**
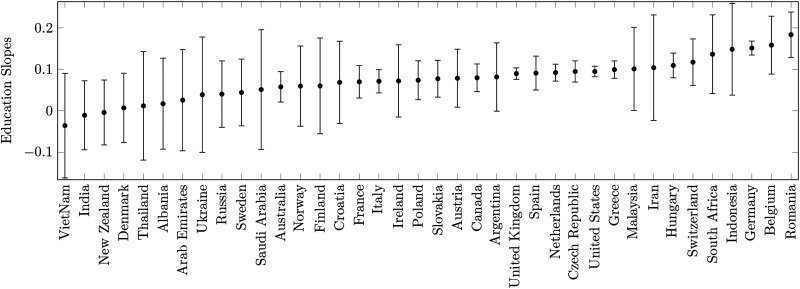
**Education effect size across countries.** Education effect sizes are the education slopes of countries in a linear mixed model predicting wayfinding performance with fixed effects for age and gender, and random effect for country, with random slopes for education clustered by country. Positive slopes correspond to a positive effect of tertiary education on wayfinding performance. Error bars correspond to standard errors.

**Figure 4.**
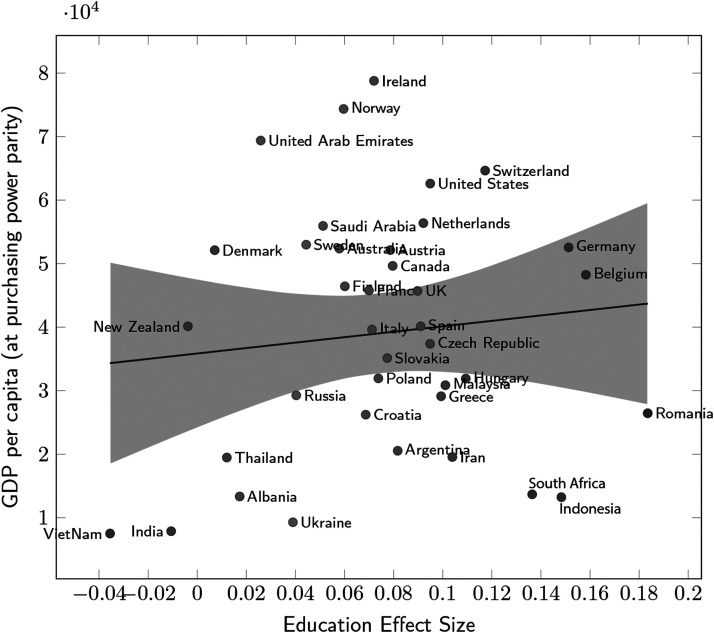
**Association between GDP per capita and education effect size.** Education effect sizes are the education slopes of countries in a linear mixed model predicting wayfinding performance with fixed effects for age and gender, and random effect for country, with random slopes for education clustered by country. Positive slopes correspond to a positive effect of tertiary education on wayfinding performance. GDP per capita is in international dollars (GDP, 2018). The shaded areas correspond to the 95% CI of the linear regression of GDP per capita on the country education slopes (*r* = 0.15, *p* = 0.38).

Figure 3 represents the education slopes (effect sizes) for each country, with positive values indicating an advantage for participants with tertiary education. The difference in standard errors is mostly driven by the difference in sample size between countries. To explore the correlates of this variation across countries we tested the relationship between education effect size and the GDP per capita (at purchasing power parity)—see Figure 4. The education effect size in each country was estimated by the LMM random slopes clustered by country, which are adjusted for the fixed effects. To show this effect in a more classic linear model we fit a linear regression to predict the wayfinding performance with the age, gender, education, country, and the interaction between education and country as predictors:

WF∼age+gender+education*country

We found a significant effect of age (*F* = 63721, *p* < 0.001), gender (*F* = 21771, *p* < 0.001), country (*F* = 188.19, *p* < 0.001), education (*F* = 1166.90) and the interaction between country and education (*F* = 2.83, *p* < 0.001).

We used GDP per capita as this indicator captures many sociodemographic trends at the country level and was strongly correlated to countries’ average wayfinding ability in previous studies (Coutrot et al., 2018; Gilles et al., 2024). However, we did not find a significant relationship between these variables (bivariate Pearson’s correlation *r*(36) = 0.11, *p* = 0.52). In term of Hedge’s *g*, the spectrum of the education effect ranged from near-zero and non-significant in India (Bayes’ factor = 0.08, Hedge’s *g* = −0.03, 95% CI = [−0.15, 0.08]), to modest and significant in Romania (Bayes’ factor = 345.44, Hedge’s *g* = 0.15, 95% CI = [0.08, 0.22]).

### Modulation by Task Difficulty

The education effect size in each level was estimated by the LMM random slopes clustered by levels, which are adjusted for the fixed effects. We used the difference between the median trajectory length and the shortest trajectory length as a proxy for the level difficulty:

$$\text{Difficulty}=\frac{\text{median}\left(TL\right)-\text{min}\left(TL\right)}{\text{min}\left(TL\right)}$$

TL is a vector containing the trajectory lengths of all participants at a given level. Given the sample size of the dataset, the shortest trajectory is near-optimal so the numerator quantifies the difference between the median and the optimal trajectory length, while the denominator normalizes by the optimal trajectory length, so that the difficulty of larger levels is not artificially inflated. Note that since SHQ difficulty was designed to increase as the player progresses in the game, difficulty and level numbers (in sequential order) are strongly correlated (bivariate Pearson’s correlation *r*(42) = 0.77, *p* < 0.001).

We tested whether the effect size of education was modulated by the task difficulty. We selected a subset of participants who completed all SHQ levels (75 levels, 10,626 participants) and fitted a LMM with fixed effects for age and gender, and random effect for level, with random slopes for education clustered by level:

$$WF∼\text{age}+\text{gender}+\left(1+\text{education}/\text{level}\right)$$

**Figure 5.**
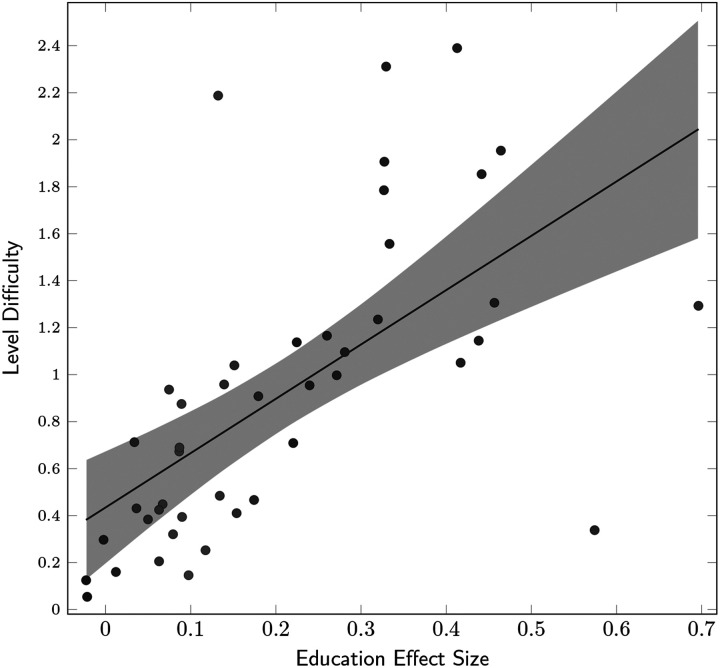
**Association between level difficulty and education effect size.** Education effect sizes are the education slopes of countries in a linear mixed model predicting wayfinding performance with fixed effects for age and gender, and random slopes for education clustered by level. This model was computed on a subset of participants who completed all SHQ levels (*N* = 10,626 participants). Positive slopes correspond to a positive effect of tertiary education on wayfinding performance. The shaded areas correspond to the 95% CI of the linear regression of level difficulty on the level education slopes (*r*(42) = 0.63, *p* < 0.001).

We included all the wayfinding levels (44 levels, since not all SHQ levels are wayfinding levels). We found a significant correlation between a level’s difficulty and the effect size of its association with education (bivariate Pearson’s correlation *r*(42) = 0.63, *p* < 0.001; see Figure 5). That is, more difficult SHQ levels tended to have a stronger association with education.

### Education and Wayfinding Ability: Correlation or Causation?

**Figure 6.**
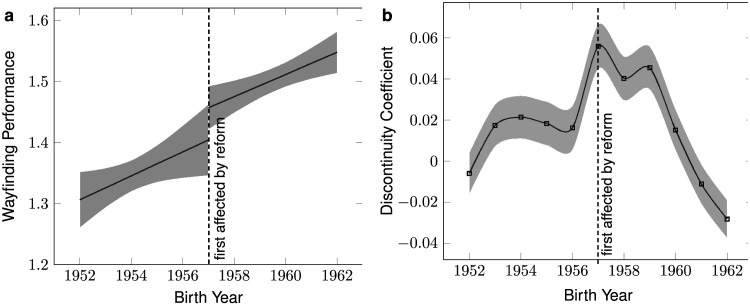
**Effect of the change of minimum school leaving age on wayfinding ability.** (a) Regression lines of birth year on wayfinding performance 5 years before (*N* = 15,902) and 5 years after (*N* = 24,627) the reform in the UK (birth year of the first cohort potentially affected by the reform = 1957). Error bars correspond to 95% CI. (b) Discontinuity coefficients from regression discontinuity designs computed in a 10 year window around 1957. Error bars correspond to standard errors.

So far, we have been concerned with correlations between wayfinding ability and education. However, these analyses are unable to assess whether differences in wayfinding ability across education groups are *caused* by differences in education, or whether instead this difference is due to other factors, such as socioeconomic differences, that are themselves correlated to education. To examine whether education had causal effects on wayfinding ability in our sample, we exploited a natural experiment, where variation in education was produced by government policies that raised the minimum school leaving age. This kind of exogenous reform allows us to focus on differences in education that are not confounded by endogenous factors (observed or unobserved characteristics of the participants in the experiment), producing a causal analysis via instrumental variables or regression-discontinuity designs (Hahn et al., 2001). Previous studies have successfully used school-leaving-age changes to investigate the causal effects of education on health and psychological outcomes, including on cognitive functions (Davies et al., 2018; Lövdén et al., 2020; Nguyen et al., 2016). Since participants only entered their home country when playing SHQ, and not finer-grained regional information, we could not include countries where the reform year varied across regions within countries in the analysis (for example, the USA and Germany; Brunello et al., 2009). We chose to focus on the United Kingdom (UK) as it was, to our knowledge, the only country that implemented a nation-wide educational reform changing the school leaving age for a high number of participants included in our sample. The relevant reform was implemented in the UK in 1972, raising the minimum school leaving age from 15 to 16 years old. The birth date of the first potentially-affected cohort was 1957 (Harmon & Walker, 1995). We included participants from the UK who were born within a 10 year window around 1957. This resulted in 41,754 participants included in the following analysis. Thus, the oldest participants included in this analysis were in their 69th year at the time of data collection (2016). In the UK, the start of the age selection bias in SHQ data is 82 years old (Gilles et al., 2024), meaning this sample is unlikely to be impacted by this bias. Figure 6a shows the regression lines of birth year on wayfinding performance calculated with participants born before (*N* = 15,902) and after (*N* = 24,627) the first cohort affected by the reform (year 1957). From the regression line based on the pre-reform participants (see Figure 6), we see that the wayfinding performance value in 1957 is: *WF* = 1.40, 95% CI = [1.35, 1.46]. This value is lower than the one given by the regression based on the participants affected by the reform: *WF* = 1.46, 95% CI = [1.42, 1.49]. The difference between these two values is Δ*WF* = 0.06. Following Ritchie and Tucker-Drob (2018), we rescaled Δ*WF* into the number of IQ point units, on the standard IQ scale (*M* = 100, *SD* = 15), associated with 1 additional year of education. To do so, we divided Δ*WF* by the standard deviation of *WF* and multiplied them by 15. This calculation gives an average increase of 0.8 IQ point unit associated with the reform, which is in the lower end of the effect sizes reported in the aforementioned meta-analysis (approximately 1 to 5 IQ points for an additional year of education).

This was *prima facie* evidence for an educational effect, but for a formal assessment of the significance of this difference, and a test of whether the effect is at its largest when the cut point is the birth year of the first cohort affected by the reform (as would be expected if education had a causal influence), we ran a regression discontinuity analysis. We used a similar design as in previous studies exploring the causal effect of education on other variables, such as health outcomes (Davies et al., 2018, 2023). We computed the following linear model:

WF∼1+birthyear+discontinuity

with *birthyear* the year of birth of the participants, and *discontinuity* a dummy variable. Let *i* ∈ [1, …, *N*], *N* the number of participants, and *c* a scalar, discontinuity(i) = 0 if birthyear(i) < *c* and discontinuity(i) = 1 if birthyear(i) ≥ *c*. We varied *c* in a 10 year window around the birth year of the first potentially affected cohort, and computed a linear regression for each *c* value. As noted above, if the reform had an effect on wayfinding performance, it should be strongest when *c* = 1957, i.e., the birth year of the first potentially affected cohort. Figure 6b shows the discontinuity coefficient for *c* values between 1952 and 1962. Results from the eleven corresponding models (from *c* = 1952 to *c* = 1962) are reported in Table 1. The largest discontinuity coefficient corresponds to *c* = 1957.

**Table 1.**
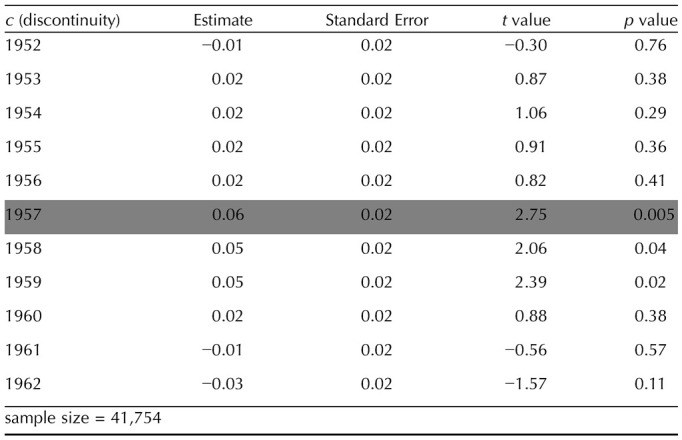
Statistics of the discontinuity parameter of 11 regression discontinuity models. The discontinuity varied between 1952 and 1962. We highlighted in black the model with the discontinuity corresponding to the birth year of the first cohort affected by the raise of the minimum school leaving age.

As a control experiment, we performed the same analysis on a variable we didn’t expect education to affect. The first two levels of the game are tutorial levels to explain the controls. Simply following the path will lead the player to the goal and no demand is made on spatial navigation ability. We defined the “performance” at these training levels as the sum of their trajectory lengths. To take into account the difference in size of these two levels, we divided each length by the length of the shortest trajectory of the corresponding level. This “training performance” value should simply reflect motor skills and is weakly correlated to wayfinding ability (Pearson’s correlation with Wayfinding Performance: *r* = 0.10). The regression discontinuity analysis on this value does not yield any significant discontinuity coefficient (the highest one is *c* = 0.02, *SE* = 0.01, *t*(41751) = 1.62, *p* = 0.11). Overall, these results support the existence of a causal relationship whereby greater education has a positive impact on wayfinding ability, at least in the UK. This discontinuity effect is likely a Local Average Treatment Effect—the effect measured when forcing people to stay in school who would otherwise have left at the earliest point (age 15). It can be different from the effect measured if everyone had been forced to stay one more year in school, even those who received tertiary education. This result echoes previous studies that used a regression discontinuity design to evidence a causal link between spatial skill instruction and improved performance both at calculus and at a mental rotation test (Sorby et al., 2013, 2018). It is also consistent with the fact that the association between STEM achievement and spatial abilities is strongest in novices, but becomes less important at a later stage of education (Kozhevnikov et al., 2007; Uttal et al., 2013).

On average, there is a significant association between education and wayfinding ability across age, gender and countries. We have shown that the strength of this association depends on the country, and that it tends to increase with age. We have also shown that there is a causal relationship between education and wayfinding ability in the UK. The mechanisms involved in this association remain unclear. Since wayfinding is not often explicitly taught in school (with the notable exception of Scandinavia, see Annerstedt, 2008; Coutrot et al., 2018), it is likely a byproduct of general cognitive improvements due to schools teaching skills that enhance cognition, e.g., problem solving (Ritchie & Tucker-Drob, 2018). Recent evidence indicates that children’s spatial skills stabilize around the age of 12 and that small scale spatial abilities do not predict spatial navigation ability (Brucato et al., 2022). More research is needed to disentangle the contributions of the different aspects of the curriculum to spatial ability, from fluid intelligence (e.g., short-term memory) to cristallized intelligence (general knowledge). The determinants of the difference in the association between education and spatial ability across countries also needs to be further examined. While wayfinding ability has been shown to be highly correlated to the Gross Domestic Product per capita (Coutrot et al., 2018), this is not the case for the effect of education. The Programme for International Student Assessment (PISA) evaluates educational systems across countries by testing 15 year old school pupils. Previous studies showed that PISA scores are good predictors of countries’ GDP per capita, but not of the average financial expenditure per student (Hunt & Wittmann, 2008; Hanushek & Woessmann, 2011). Unfortunately we cannot use PISA scores in our analysis as we measured the added value of tertiary over secondary education, and not students’ attainment at 15 years old. Similar worldwide scores measured at different levels of education would be key to further understand these differences across countries. The link between precise measures of educational attainment and spatial navigation ability could also be disentangled with longitudinal studies, or birth cohort such as the 1970 British Birth Cohort (Flouri, 2006).

## METHODS

### Informed Consent and Ethics Approval

This study has been approved by the UCL Ethics Research Committee. The ethics project ID number is CPB/2013/015. Participants were explained the purpose of the game when opening the app, before they could start playing. Players had to tick an ‘opt in’ box if they agreed to share their data with us. They were guided to the settings where the opt out option always remained available. They could also choose to provide their demographic or not. This was done in two steps. First, they could enter their age, gender and home country. Then, after having played a few levels, participants could provide further information such as their average sleep duration, level of education, commute duration, and the type of environment they grew up in.

### Navigation Task

Sea Hero Quest is an app available on smartphones and tablets. It was freely available on the App Store and Google Play between May 2016 and March 2019. At the beginning of each wayfinding level, participants were asked to memorize the locations of 1 to 5 checkpoints to visit on a map. The map disappeared, and they had to navigate a boat through a virtual environment to find the different checkpoints in a set order (Figure 1). Participants were incentivized to complete the task as quickly as possible; they were awarded ‘stars’ when finishing each level before a set time. The game was manipulated through four simple controls: tap left to turn left, tap right to turn right, swipe up to speed up and swipe down to halt. The experimental tasks in SHQ were accessed by unlocking levels sequentially. These levels comprised 5 themed areas, each containing 9 wayfinding levels. See Coutrot et al. (2018) for more details.

### Participants

A total of 3,881,449 participants played at least one level of the game. 60.8% of the participants provided basic demographics (age, gender, nationality) and 27.6% provided more detailed demographics (type of environment they grew-up in, level of education …). The inclusion criteria were the same as in Coutrot, Manley, et al. (2022). First, to provide a reliable estimate of spatial navigation ability, we only included participants who had completed a minimum of eleven levels of the game (including 4 wayfinding levels), and who entered all their demographics. Then, we removed participants above 70 years old because we previously showed a strong selection bias in this group causing their performance to be substantially higher than would be expected in unselected participants of the same age (Coutrot et al., 2018). Finally, we removed participants from countries with fewer than 500 players, or with the education classes more than 10-fold imbalanced. This resulted in 397,162 participants from 38 countries included in our analysis. Among them, there were 212,143 males (mean age: 37.81 ± 13.59 years old) and 185,173 females (mean age: 38.67 ± 14.92 years old). The levels of education were: university (42%, *N* = 166,714), college (28%, *N* = 111,463), high-school (27%, *N* = 107,849), and no formal (3%, *N* = 11,290). We merged ‘university’ and ‘college’ into a unique ‘tertiary education’ level (70%, *N* = 278,177). Similarly, we merged ‘high-school’ and ‘no formal’ into a unique ‘secondary education and lower’ level (30%, *N* = 119,139). We chose not to analyse separately the ‘no formal’ group due to its relative low sample size.

### Performance Metric

To quantify wayfinding ability, we defined the “wayfinding performance” metric (*WF*), which captures how efficient participants were in completing the wayfinding levels, while correcting for video-gaming skills. As in Coutrot et al. (2018) and Coutrot, Manley, et al. (2022), we computed the trajectory length in pixels, defined as the sum of the Euclidean distance between the points of the trajectory. The coordinates of participants’ trajectories were sampled every 500 ms. Shorter trajectories correspond to good performances, while longer ones indicate that participants followed sub-optimal routes. To control for video gaming skills, we divided the trajectory length of each level by the sum of the trajectory lengths of the first two tutorial levels, which did not require any wayfinding ability. *WF* was defined as the 1st component of a Principal Component Analysis across the normalized trajectory lengths of the first 4 wayfinding levels (levels 6, 7, 8, and 11). We used the data from the first four wayfinding levels as it provides a good trade-off between robustness (the more levels we include, the more robust the measure) and sample size (the number of players decreases with the number of the level). Using the first component of a PCA has several advantages. First it normalizes the trajectory lengths, putting the different levels on a same scale. This is important because the levels vary in size, but larger levels are not necessarily harder and should not overcontribute to the performance metric. Second it creates a variable that maximizes the variance in the dataset, which remains highly interpretable, as it is simply based on a linear combination of the trajectory lengths of the different levels.

### Linear Mixed Models

The parameters of the linear mixed models have been estimated with the maximum likelihood method, and the covariance matrix of the random effects have been estimated with the Cholesky parameterization.

## ACKNOWLEDGMENTS

The Sea Hero Quest initiative has originally been funded and supported by Deutsche Telekom. The video-game company Glitchers designed and produced the game.

## FUNDING INFORMATION

The Sea Hero Quest initiative has originally been funded and supported by Deutsche Telekom. The video-game company Glitchers designed and produced the game. Alzheimer Research UK (ARUK-DT2016-1-HS MH) funded the analysis.

## AUTHOR CONTRIBUTIONS

H. S.: Conceptualization; Funding acquisition; Investigation; Project administration; Resources; Supervision; Writing – review & editing. M. H.: Conceptualization; Funding acquisition; Project administration; Supervision; Writing – review & editing. J. W.: Conceptualization; Methodology; Writing – review & editing. C. H.: Conceptualization. R. D.: Conceptualization; Writing – review & editing. A. C.: Data curation; Formal analysis; Investigation; Methodology; Software; Validation; Visualization; Writing – original draft; Writing – review & editing. E. M.: Methodology. S. R.: Formal analysis; Methodology; Writing & review & editing. R. K.: Formal analysis; Methodology; Writing – review & editing.

## DATA AND CODE AVAILABILITY STATEMENT

The data and Matlab code (R2018a) necessary to reproduce the results presented in this manuscript are available at https://osf.io/hr93f/?view_only=65f3b141abfc41449cd24f42a0706453. We also set up a portal where researchers can invite a targeted group of participants to play Sea Hero Quest and generate data about their spatial navigation capabilities. Those invited to play the game will be sent a unique participant key, generated by the Sea Hero Quest system according to the criteria and requirements of a specific project (https://dash.seahero.quest/). Access to the portal will be granted for noncommercial purposes.

## Supplementary Material


