# Supplementary material for: Education Is Positively and Causally Linked With Spatial Navigation Ability Across the Lifespan
Source: Open Mind (Camb). 2025 Jul 26;9:926–39. doi: 10.1162/opmi.a.13 (PMC12373451; doi:10.1162/opmi.a.13)
Supplement: Supplementary file 1 [file opmi-09-926-s001.pdf]

Supplementary Information

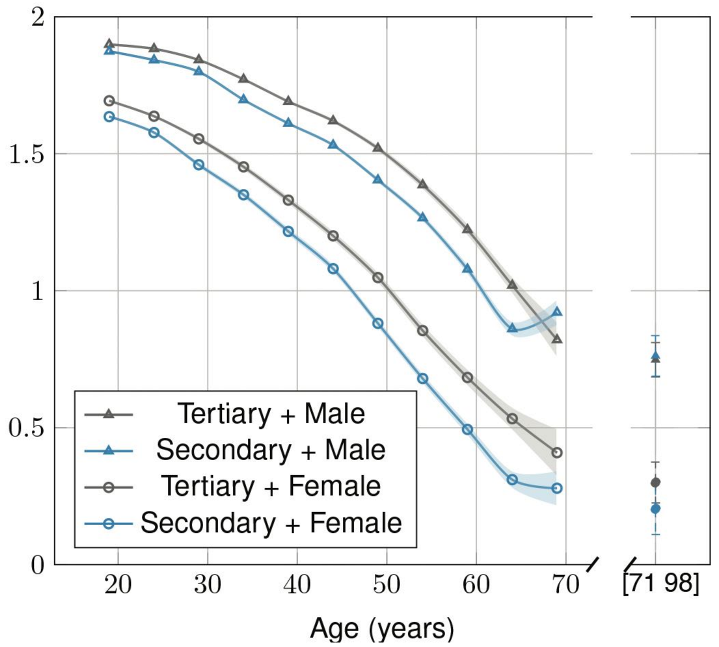

Fig. S 1. Effect of education on Wayfinding Performance - Interaction with age and gender (older participants included) Wayfinding Performance has been averaged within 5-year windows between 19 and 70 years-old. Participants between 71 and 98 years-old have been collapsed into a single data point due to the low sample size and the strong selection bias (see main text). Error bars correspond to the 95% confidence intervals.

Supplementary Analysis: Effect of the four levels of education

A multivariate linear regression was calculated to predict wayfinding performance based on age, gender, education, and their interactions. We then used an ANOVA to summarize the main effects of the regression. Age ranged from 19 to 70 years, we used 2 categories for gender (males, females), and 4 for education (no-formal, high-school, college and university). Age had the strongest association with performance ($F_{1,397154} = 41225$, $p < 0.001$), followed by gender ($F_{1,397154} = 10502$, $p < 0.001$), and education ($F_{1,397154} = 411.36$, $p < 0.001$). There was a significant interaction between age and gender ($F_{1,397154} = 1540.30$, $p < 0.001$), between age and education ($F_{1,397154} = 23.87$, $p < 0.001$), between gender and education ($F_{1,397154}$ =21.22, $p <$

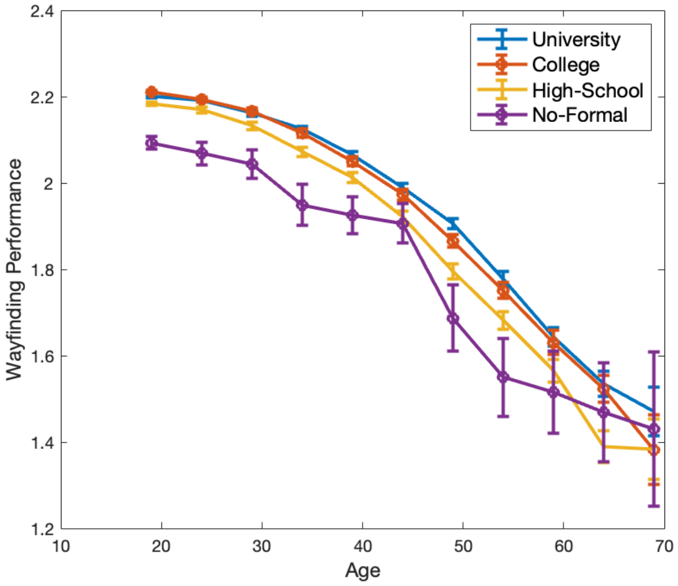

Fig. S 2. Effect of the four levels of education on Wayfinding Performance across age. Wayfinding Performance has been averaged within 5-year windows between 19 and 70 years-old. Error bars correspond to the 95% confidence intervals.

0.001), and between age, gender and education ($F_{1,397154}$ =9.87, $p = 0.001$). These interactions indicate that the effects of gender and education on wayfinding ability both increase with age, and that the effect of education is stronger in females. Looking more specifically at the contrasts between the 4 education levels ('university' being the baseline, see Figure S2 and Table S1), we notice that the difference between the 'university' level and 'high-school' level increases with age, but not between 'university' and 'no-formal'. The 3-way interaction between age, gender and education is significant and represented in Figure S3. The difference between men and women increases with age for all education levels, and this increase is more pronounced for the high-school and no-formal education levels. The results of the "no-formal" education level needs to be interpreted with caution as they only represent 3% of the participants."

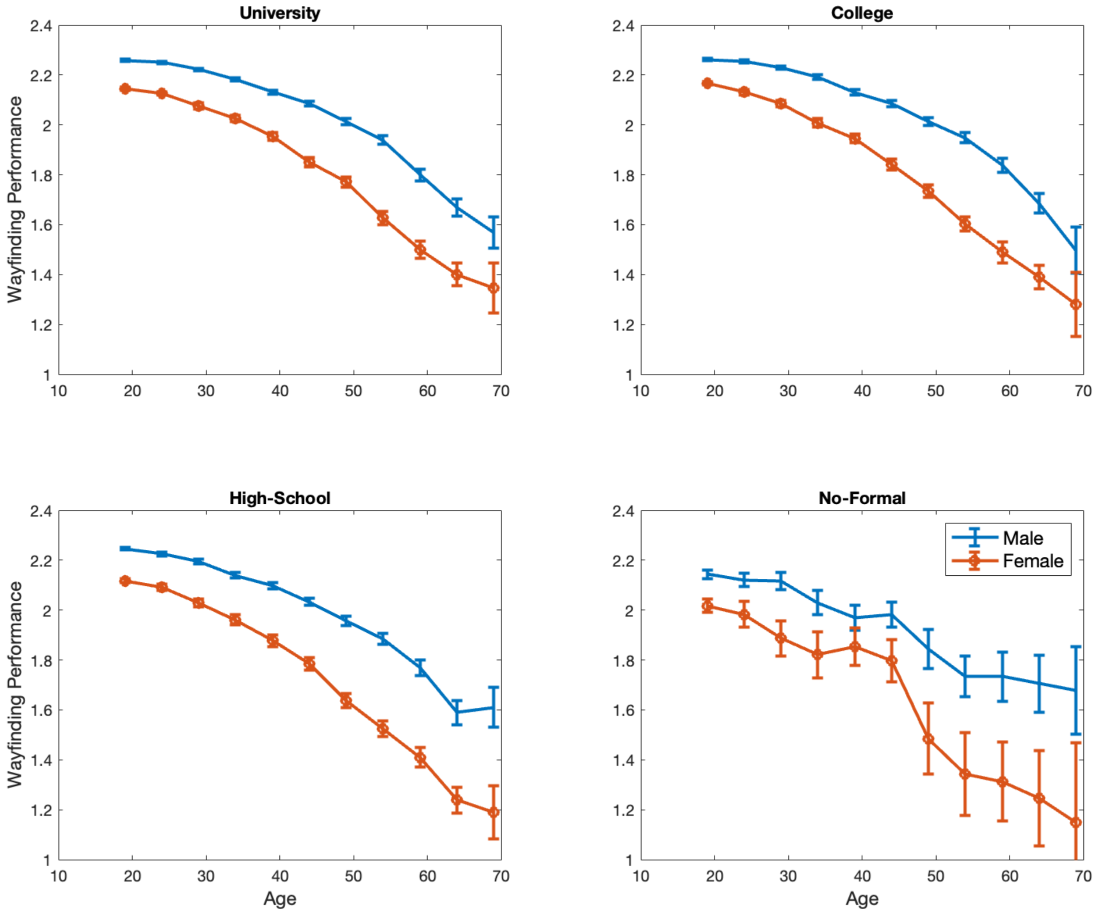

Fig. S 3. Effect of the four levels of education and gender on Wayfinding Performance across age. Wayfinding Performance has been averaged within 5-year windows between 19 and 70 years-old. Error bars correspond to the 95% confidence intervals.

```
Linear regression model:
    WF ~ 1 + age*gender + age*education + gender*education + age:gender:education

Estimated Coefficients:
```

	Estimate	SE	tStat	pValue
(Intercept)	2.5088	0.0043969	570.59	0
age	−0.014073	0.00010833	−129.91	0
gender_f	0.006219	0.0043969	1.4144	0.15724
education_college	0.046637	0.0057816	8.0665	7.2514e−16
education_high-school	0.031074	0.0057833	5.373	7.7479e−08
education_no-formal	−0.11931	0.011633	−10.256	1.1212e−24
age:gender_f	−0.0029785	0.00010833	−27.495	2.8193e−166
age:education_college	9.3699e−05	0.00014229	0.65852	0.5102
age:education_high-school	−0.00096705	0.00014138	−6.8402	7.9216e−12
age:education_no-formal	0.00045433	0.00028692	1.5835	0.11331
gender_f:education_college	0.0014908	0.0057816	0.25786	0.79651
gender_f:education_high-school	−0.0010579	0.0057833	−0.18293	0.85486
gender_f:education_no-formal	0.013371	0.011633	1.1494	0.25037
age:gender_f:education_college	0.00013481	0.00014229	0.94747	0.3434
age:gender_f:education_high-school	−0.00014935	0.00014138	−1.0564	0.29078
age:gender_f:education_no-formal	−0.00065517	0.00028692	−2.2834	0.022406

Table. S 1. Regression coefficients with wayfinding performance as the outcome and age, gender, and education (4 levels) as the predictors. ’male’ is the baseline level for gender, ’university’ is the baseline level for education.
